# Correction: The assessment of fundus image quality labeling reliability among graders with different backgrounds

**DOI:** 10.1371/journal.pone.0292776

**Published:** 2023-10-05

**Authors:** Kornélia Lenke Laurik-Feuerstein, Rishav Sapahia, Delia Cabrera DeBuc, Gábor Márk Somfai

There are errors in the Funding section. The correct Funding statement is: This study was supported in part by the Finker Frenkel Legacy Foundation (DCD), a NIH Center Grant No. P30-EY014801 to the University of Miami (DCD), and by an unrestricted grant to the University of Miami from Research to Prevent Blindness, Inc. (DCD). The project described was also supported in part by Grant Number UL1TR002736, Miami Clinical and Translational Science Institute, from the National Center for Advancing Translational Sciences and the National Institute on Minority Health and Health Disparities. Its contents are solely the responsibility of the authors and do not necessarily represent the official views of the NIH.
