# Supplementary material for: The assessment of fundus image quality labeling reliability among graders with different backgrounds
Source: PLoS One. 2022 Jul 26;17(7):e0271156. doi: 10.1371/journal.pone.0271156 (PMC9321443; doi:10.1371/journal.pone.0271156)
Supplement: S1 File — (PDF) [file pone.0271156.s001.pdf]

AI Grading Tutorial

Version 2.2, January 15, 2022.

Dear Participant,

We would like to cordially thank you for your time and effort to help us with this small project.

Our goal is to test the feasibility and performance of a simple tool for the human grading of retinal images that will be used to train a deep learning algorithm for screening purposes.

The following slides will guide you through your task and explain

- Information of the human retina
- The function of the tool itself
- and the grading methodology

It is important that you have no stress, you need to work relaxed ☺

You will be asked to grade 200 retinal images

The aim is to assess the quality of each image and group them accordingly

The application will **measure the time needed** for the grading, therefore, we kindly ask you to **do the grading possibly in one run** – although there is an option to **stop the timer in case it is necessary**.

A very brief overview of ocular anatomy…

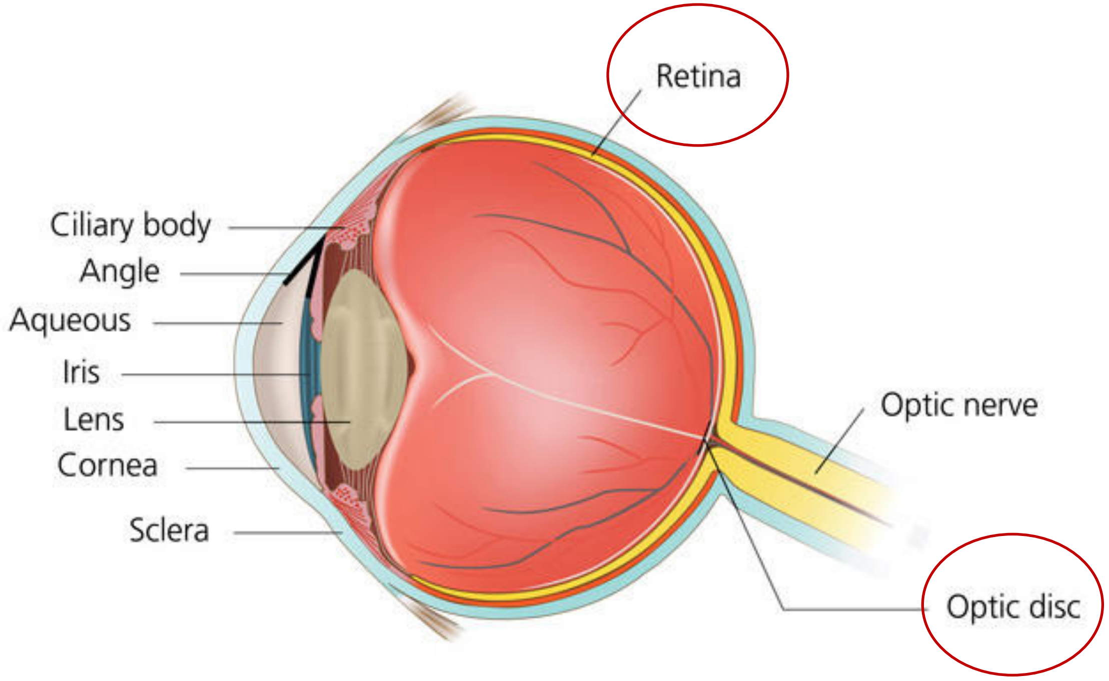

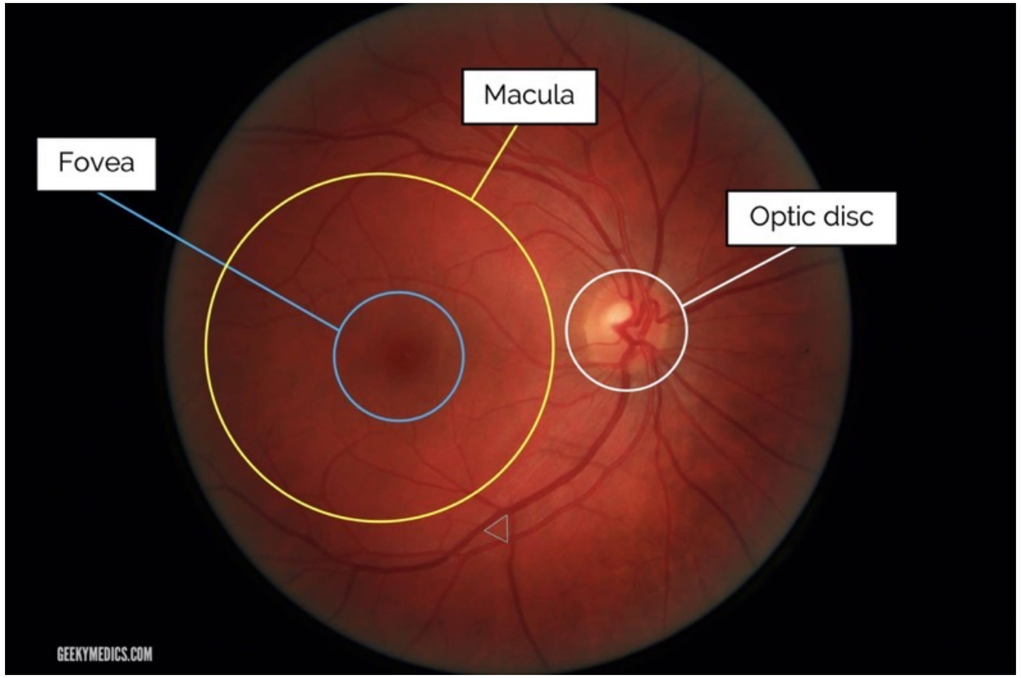

A very brief overview of retinal anatomy...

- A right eye (the fovea X is to the right compared to the optic disk O) and a left eye (the fovea is X to the left compared to the optic disk O)

Here is the outer part (called «temporal»)

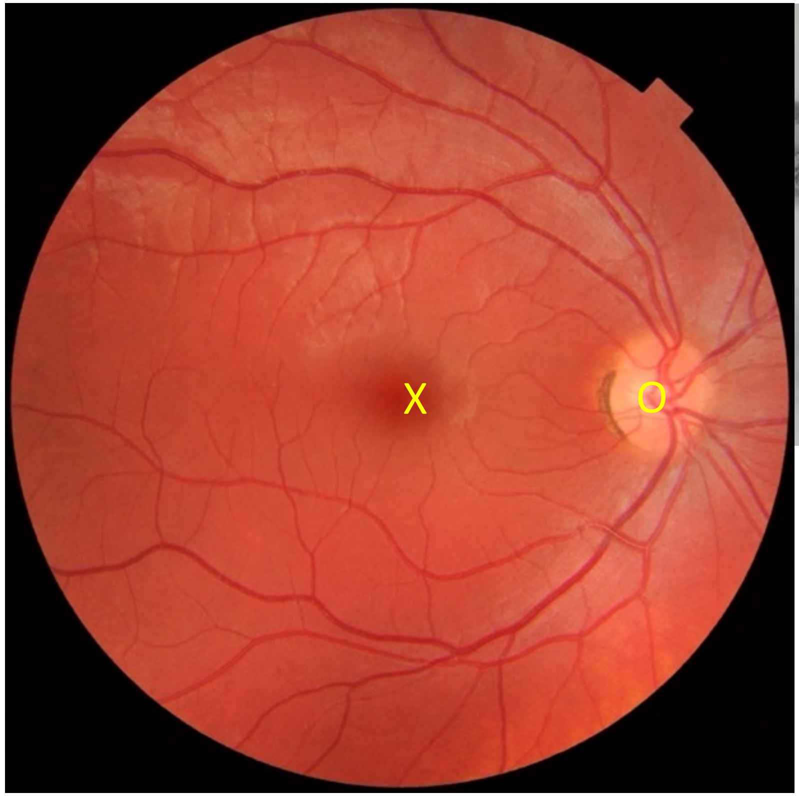

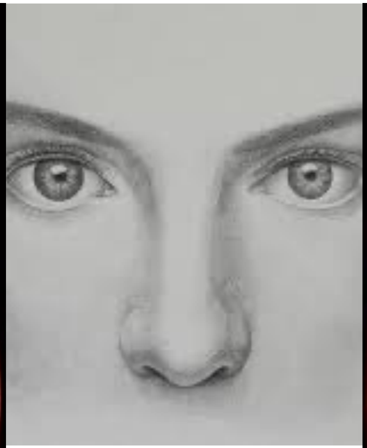

Here is the nose (this side is called «nasal»)

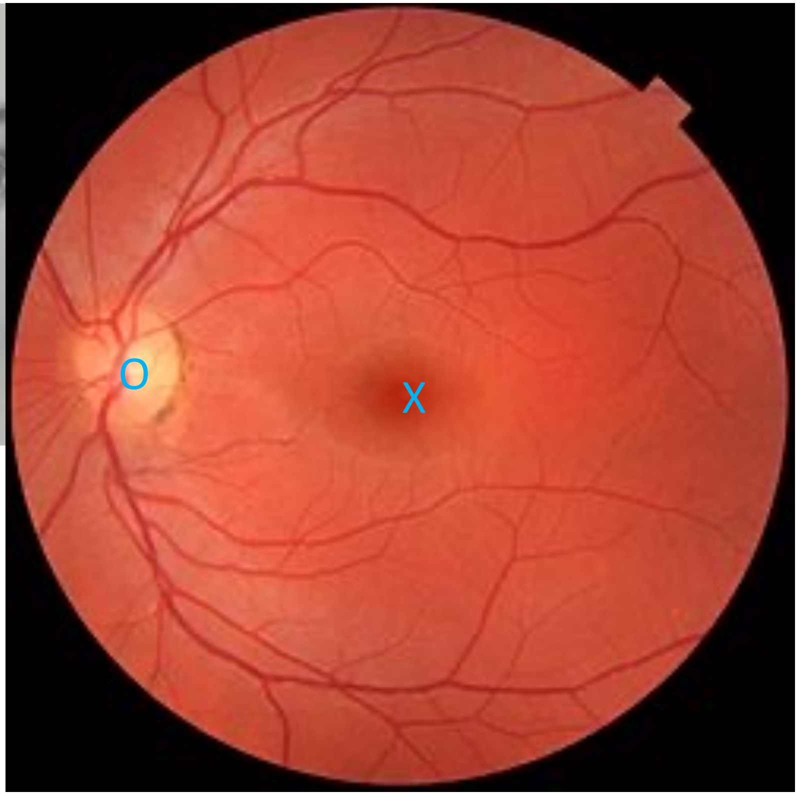

Here is the outer part (called «temporal»)

For grading you need to do the following

1. Examine each image for the 4 image quality factors:

1. Focus
2. Illumination
3. Image field definition
4. Artefacts

2. Select the correct classification:

1. Excellent – all image quality factors are optimal
2. Good – 1-2 image quality factors are not optimal
3. Adequate – 3-4 image quality factors are not optimal
4. Insufficient – at least 50% of the picture is inadequate for grading retinal lesions

Take a good look. This image is...

1. Optimally focused
2. Well lit
   - the optic disk(O) is optimally exposed
3. Well centered
   - the fovea is in the middle (x) and
   - there is an area visible temporal to the macula, on the left side
   - there is an area visible next to the optic disk(O) on the right, nasal side
4. Free of artefacts
   - the edges are well visible (no small pupil, eyelashes or arc defects)
   - there are no camera artefacts (flare, central reflex, fingerprint, dust spots)

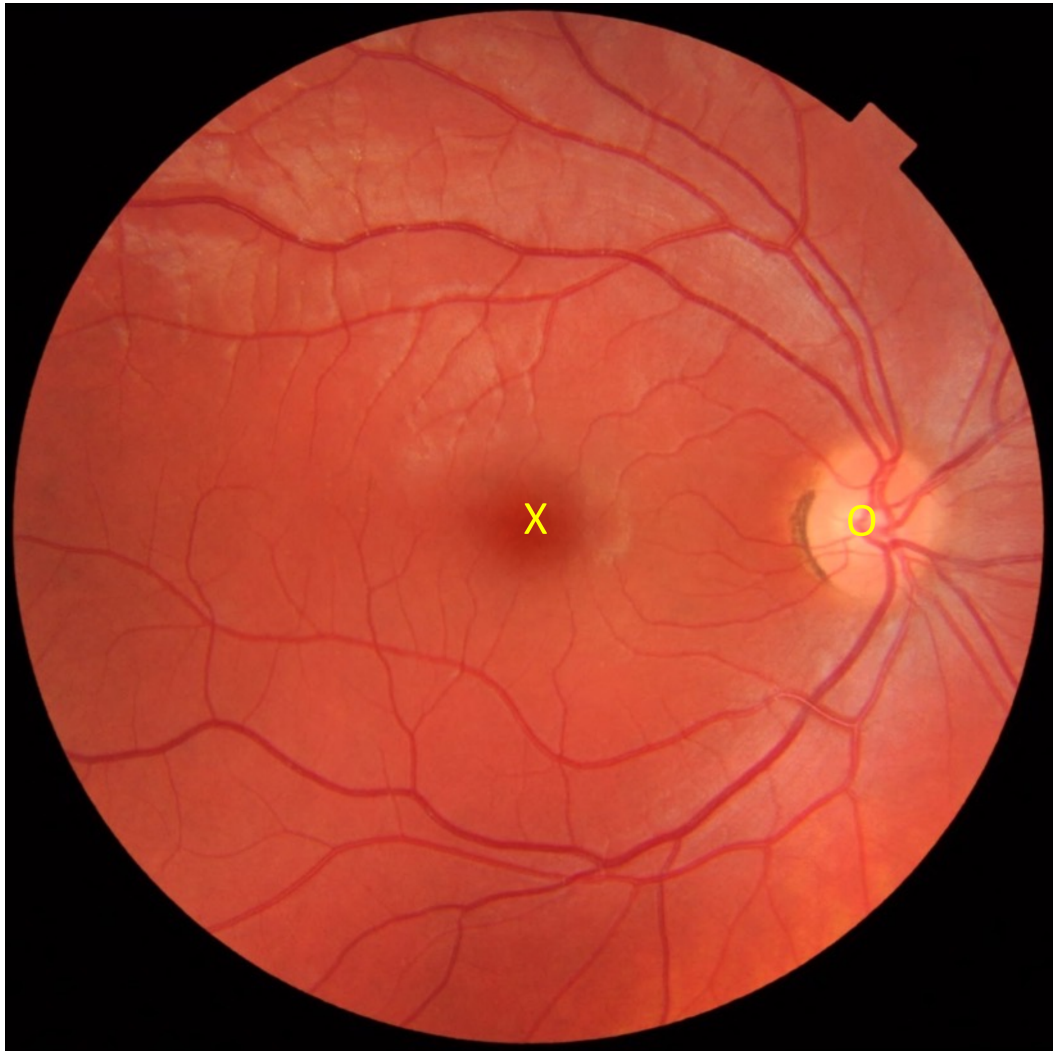

1.1. Examine the focus

Is the focus good enough for grading smaller retinal alterations (e.g. microaneurysms, venous beading or intraretinal microangiopathy)? Are small retinal vessels approximately one optic disk diameter around the fovea depicted sharply?

2.1 Grade the image

Give a score for the correct, sharp focus (0) or lack thereof (1).
If third generation branches within one optic disk diameter near the fovea can not be identified, the images should be considered insufficient.

Focus – too unsharp on the right

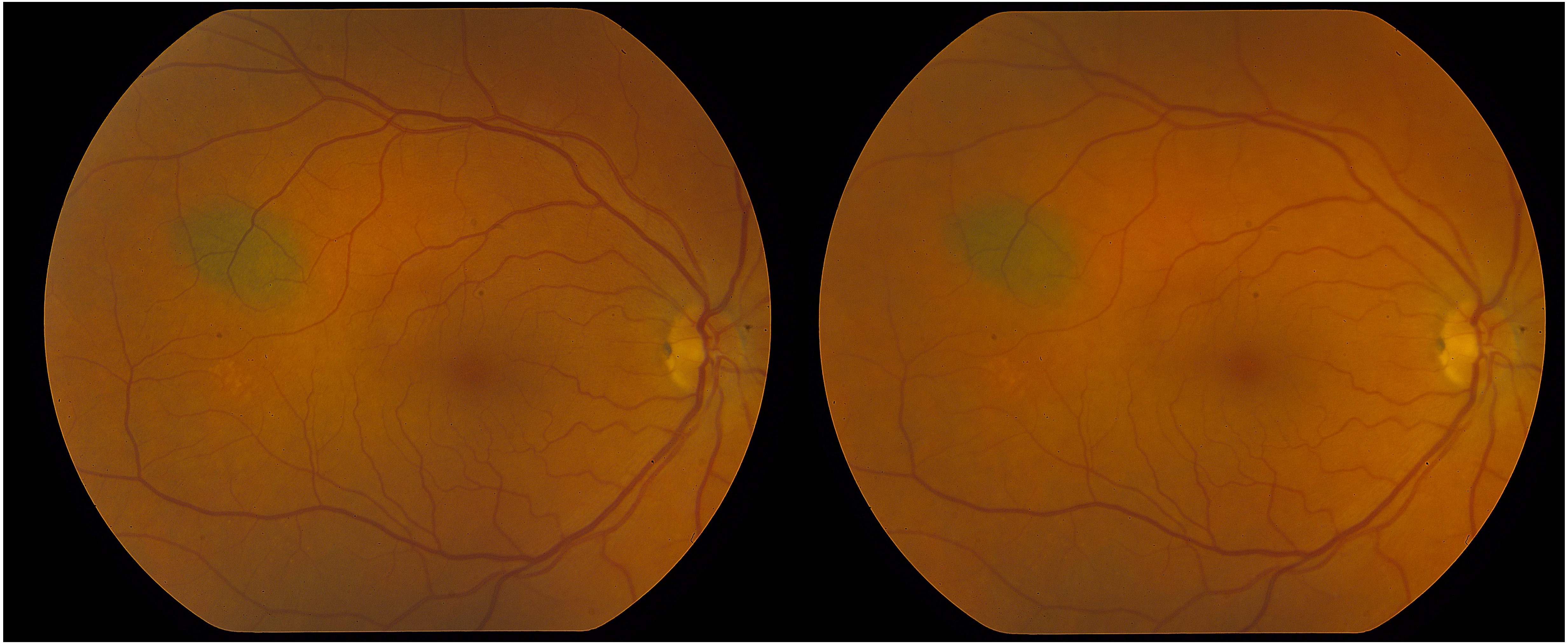

Focus – too unsharp on the right

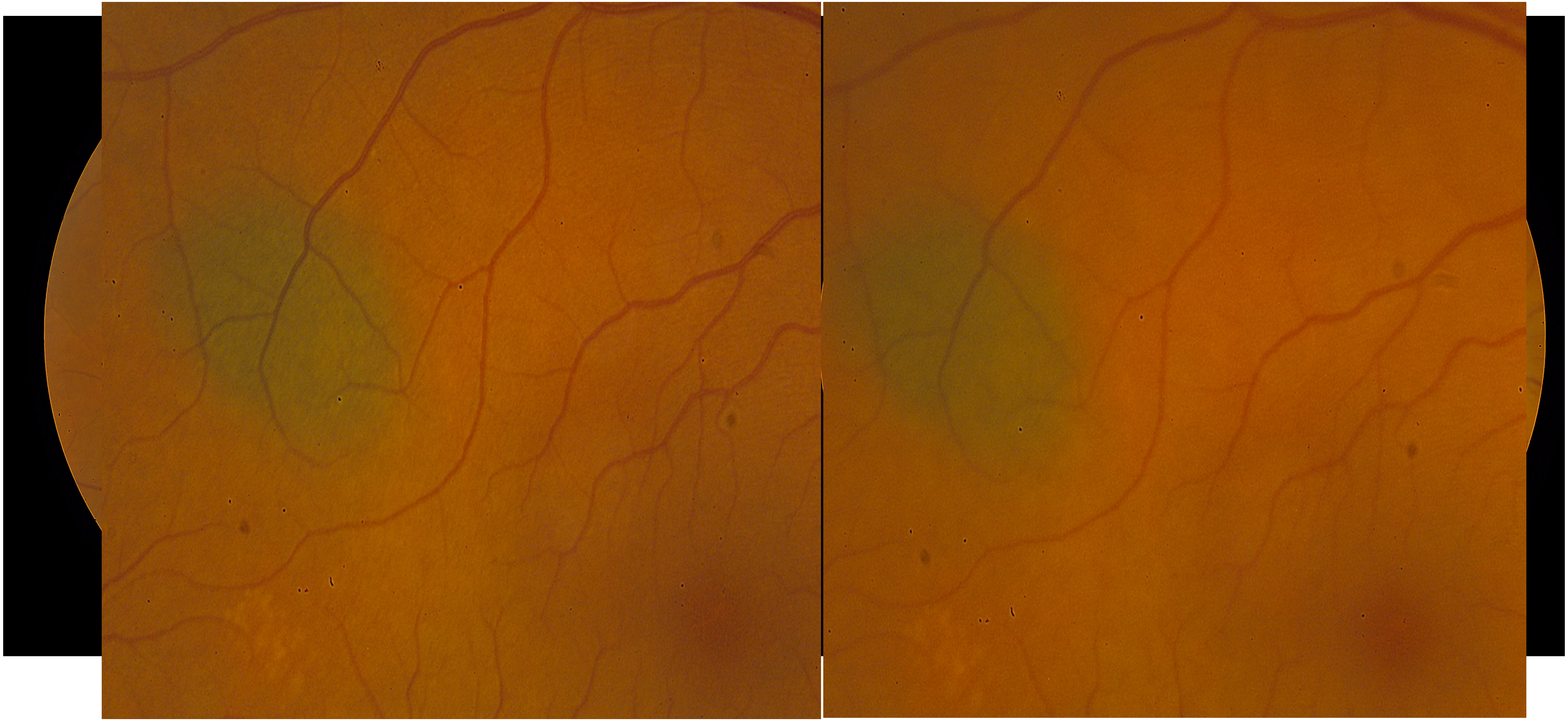

1.2. Examine the illumination

Is the image too dark, or too light – overexposed? Are there dark areas or washed-out areas that interfere with detailed grading?

2.2. Grade the image

Give a score for the well lit and optimally exposed image (0) or the too dark or too light image (1).

Optimal pictures – both images are sharp, well lit, Optic nerve head and macula well-centered, without artefacts

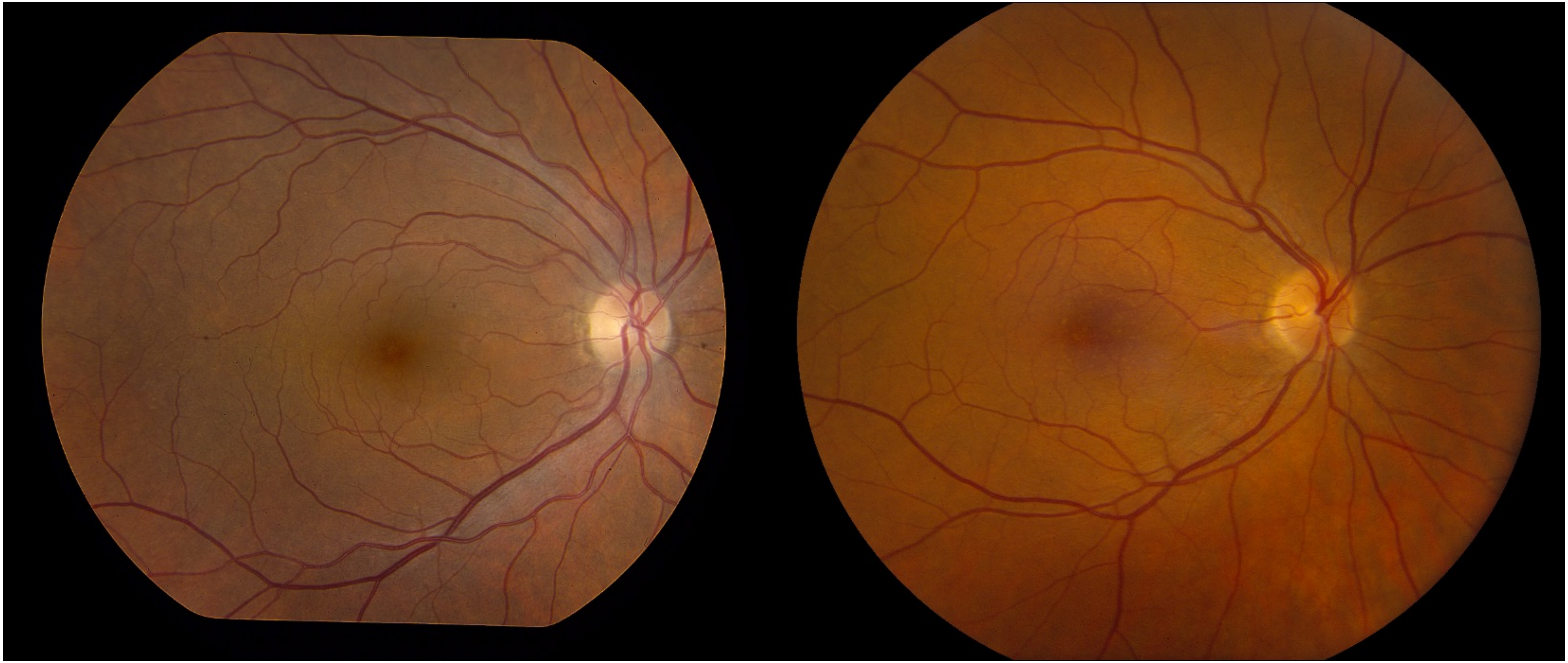

Illumination – too dark

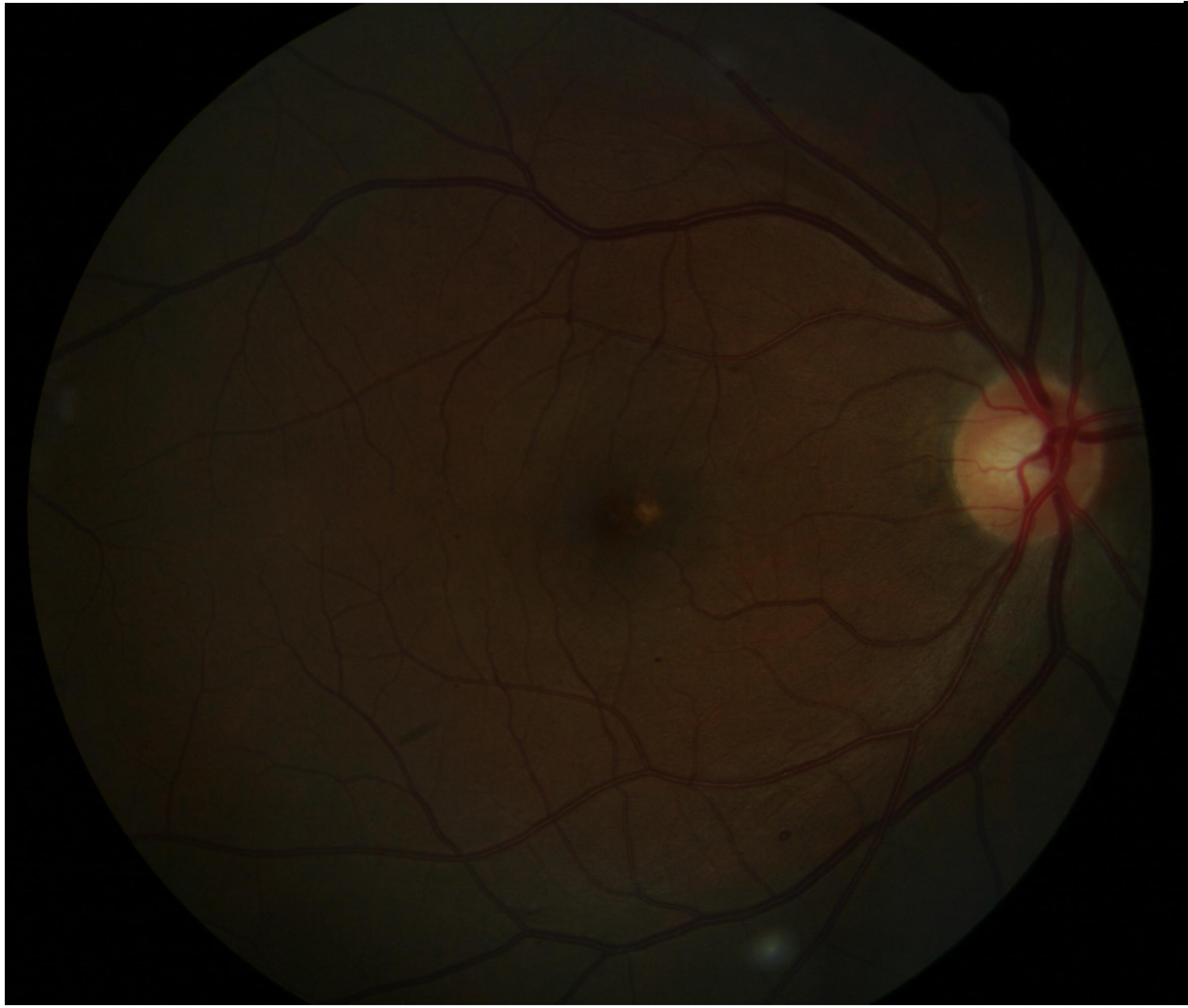

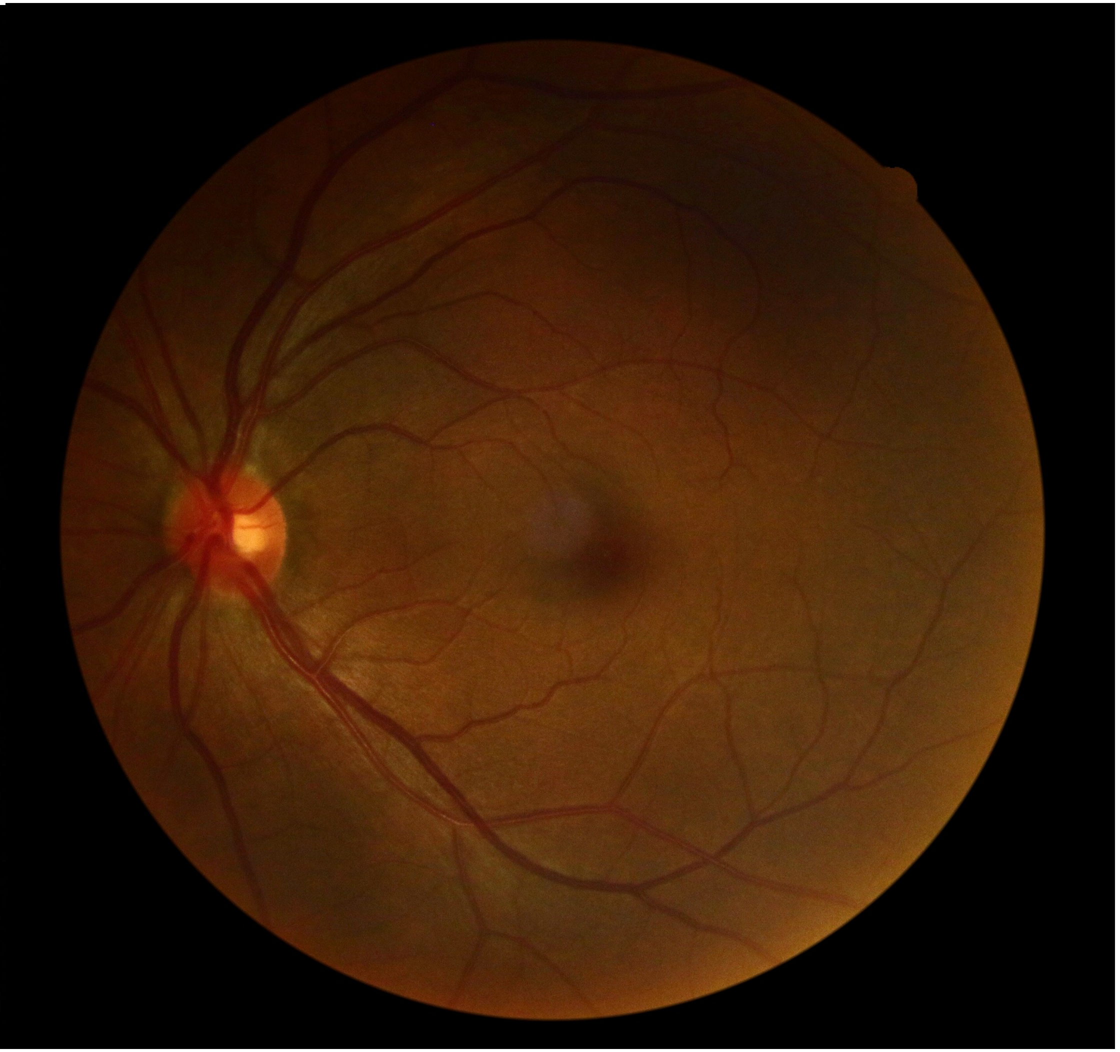

Illumination – too bright, overexposed

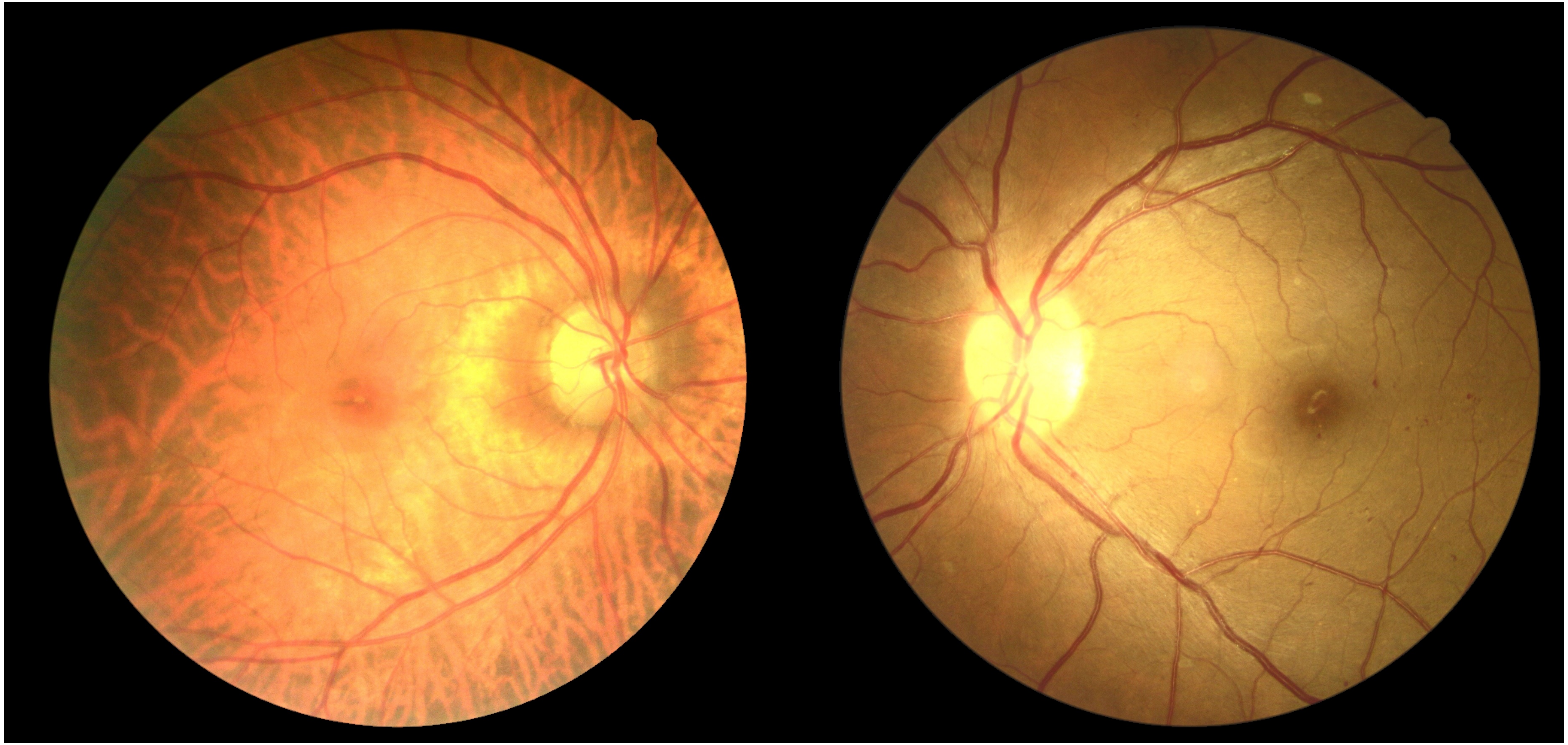

1.3. Examine the image field definition

Does the primary field include the entire optic nerve head and macula? Are the nasal (sideways from the fovea) and temporal (sideways from the optic nerve head) fields adequately centered?

2.3. Grade the image

Give a score for the optimally centered image (0) or for the images not including the total macula or even fovea or the optic disk (1).

Image field definition – the picture in the middle is optimal, the one on the left too far temporal, the one on the right too far nasal

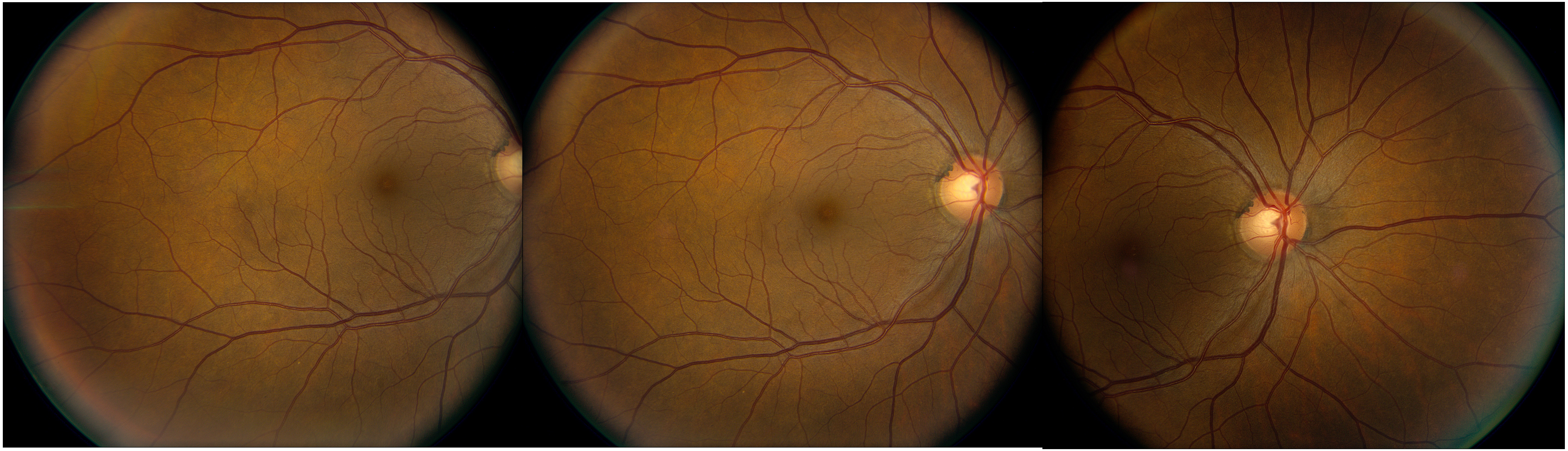

1.4. Examine the artefacts

Is the image sufficiently free of artefacts (e.g. dust spots, arc defects, flashes, fingerprints or camera reflexes and eyelash images) to allow adequate grading?

2.4. Grade the image

Give a score for the artefact free image (0) or for any artificial structural misinterpretations (1).

Artefacts

- dust spots

- lashes

- arc defects

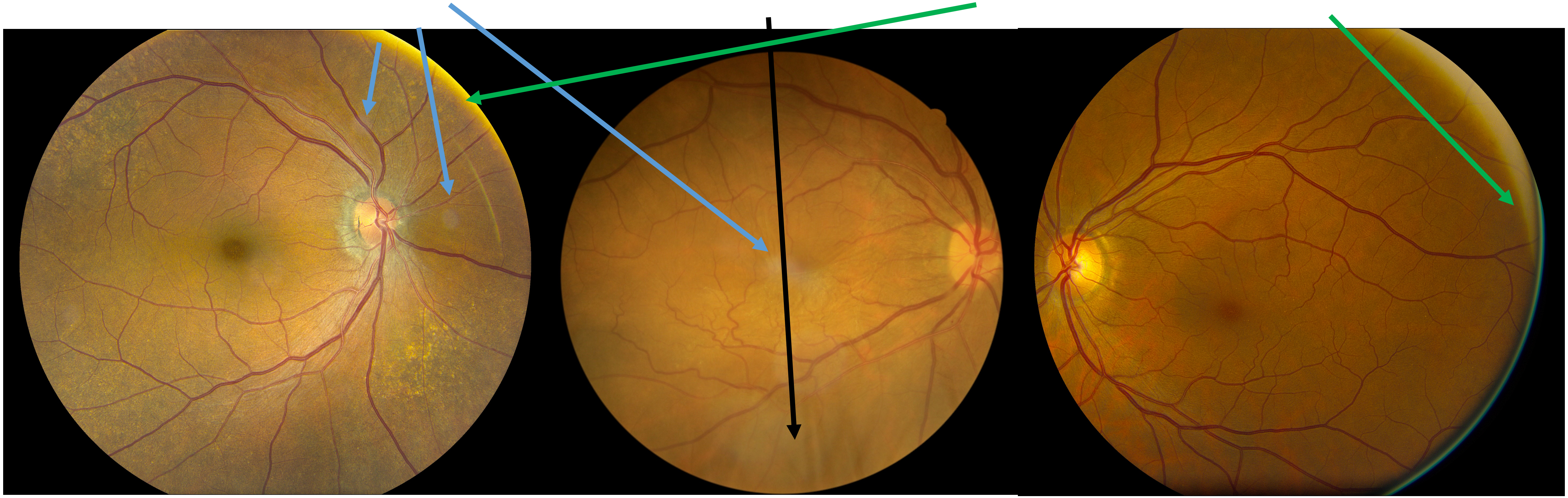

Artefacts

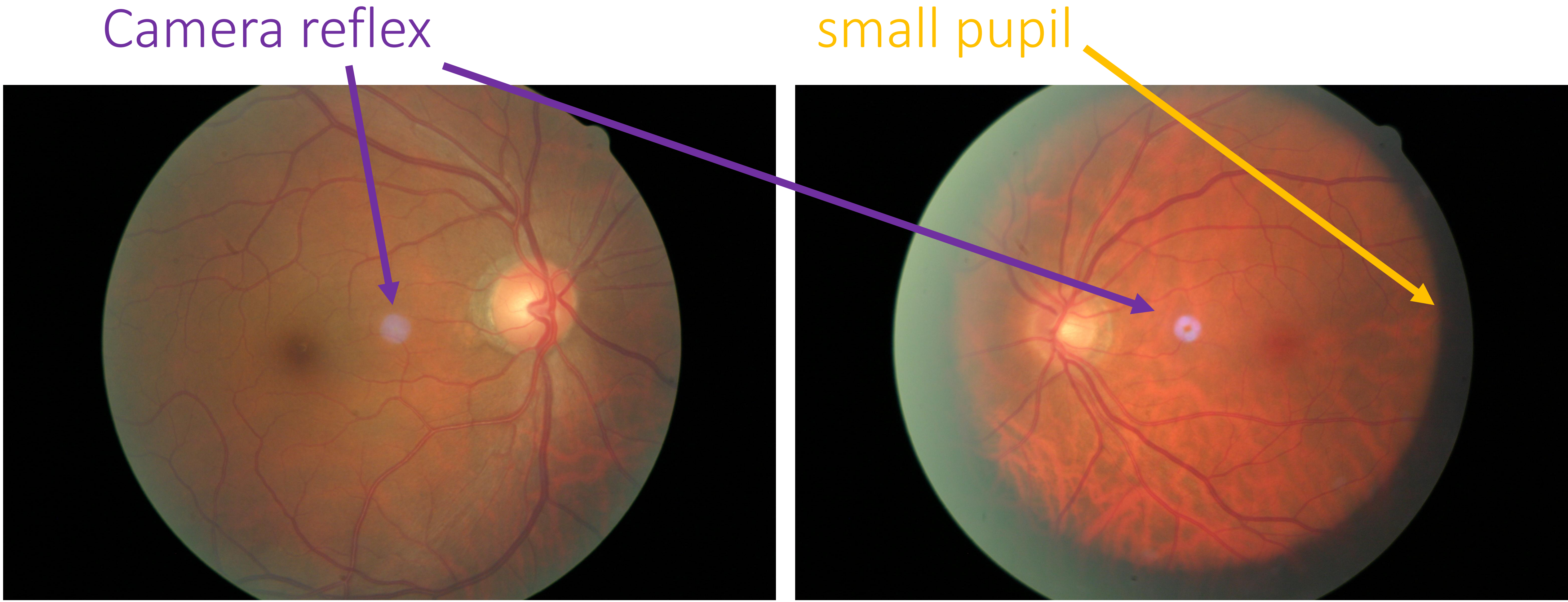

3. Finish grading – choose a category

1. **Excellent:** All image quality factors are optimal. All retinopathy lesions gradable.
2. **Good:** Problems with 1-2 of the image quality factors. All retinopathy lesions gradable.
3. **Adequate:** Problems with 3-4 of the image quality factors. All retinopathy lesions gradable.
4. **Insufficient:** One or more retinopathy lesions cannot be graded.
   - If third generation branches within one optic disk diameter near the fovea and the optic nerve head cannot be identified, the images should be considered inadequate for grading.
   - Images lacking visibility of more than 50% of the depicted retinal field should be considered inadequate for grading.

Excellent (0 problems)

sharp, well lit, well centered image, no artefacts

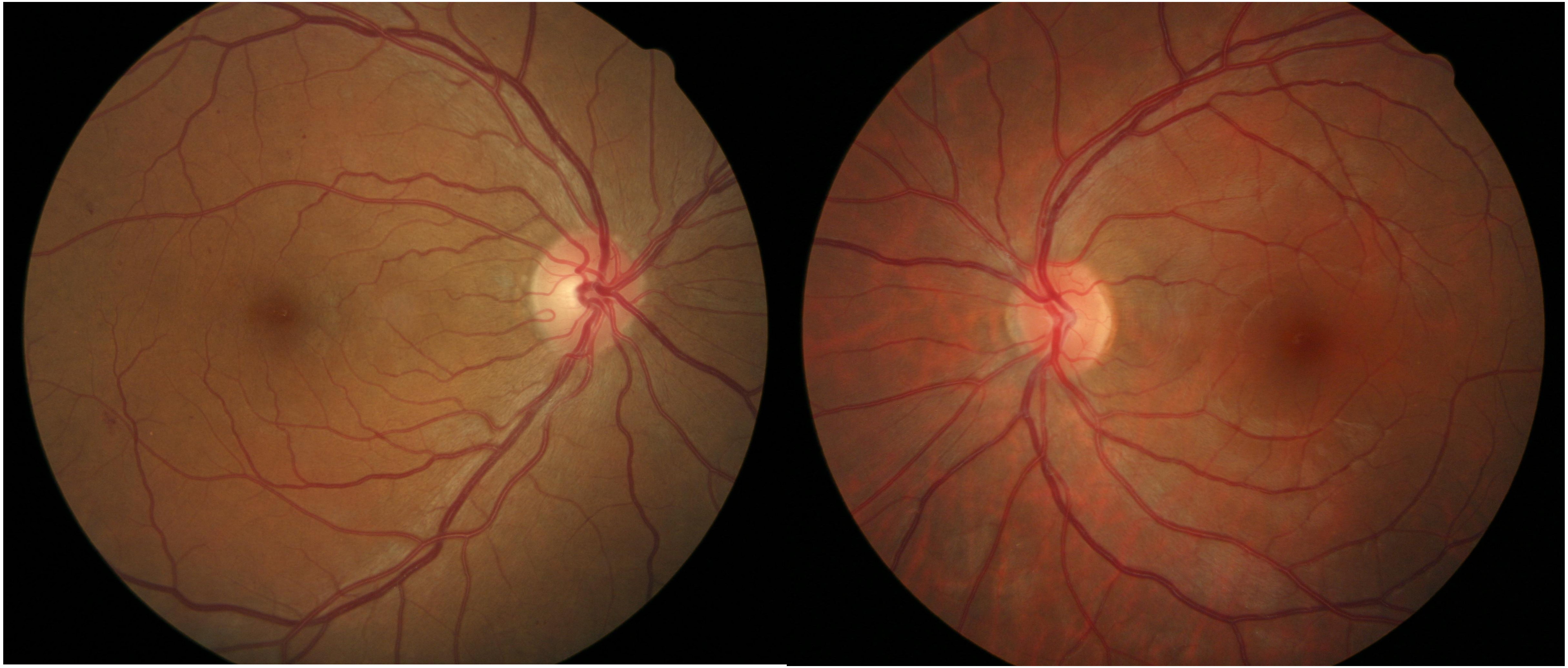

Good (1-2 problems)

Left: sharp, well lit, **image field far too nasal, minor arc defect on the right side** (2)

Right: **unsharp**, well lit, image well centered, **camera reflex and small pupil defect** (2)

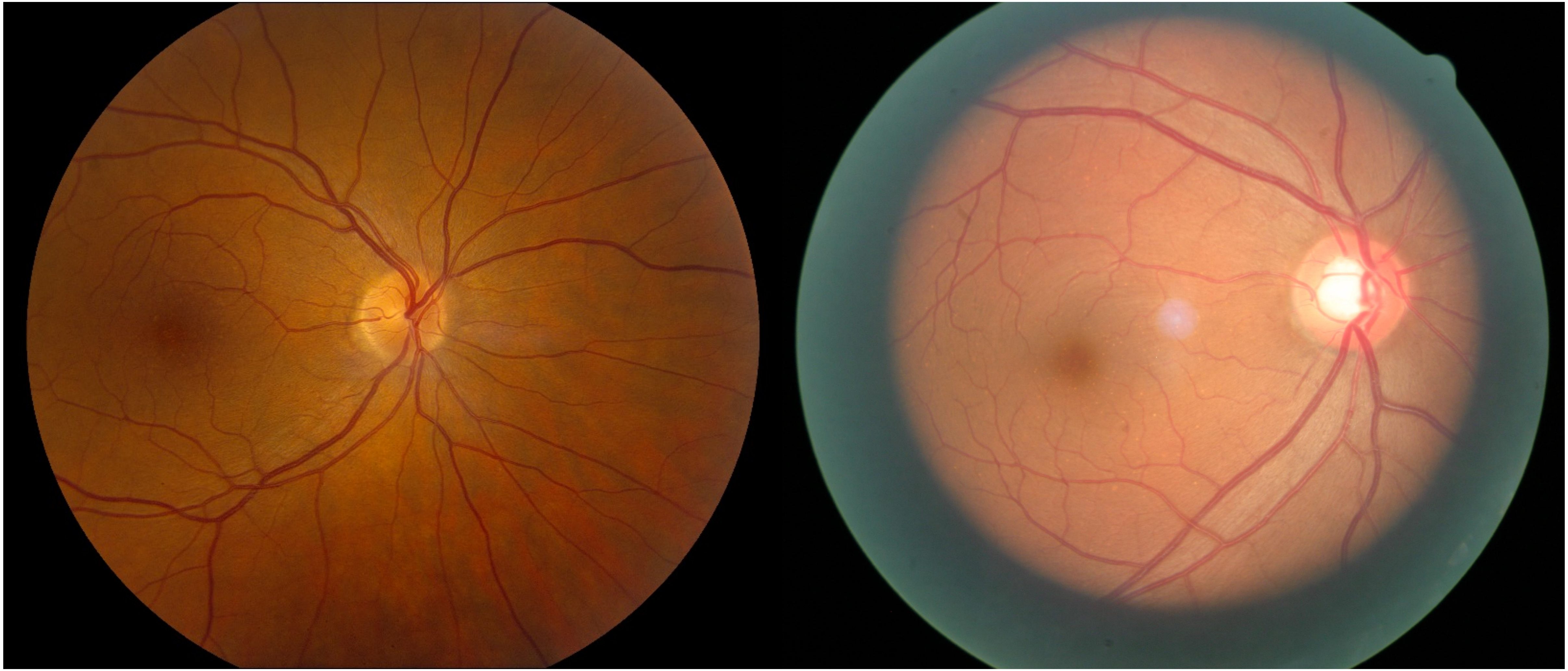

Adequate (3-4 problems)

Middle: focus ok, **too dark, image field too temporal, arc defect** (3)
Left: **unsharp,** illumination ok, **image field too temporal, arc defect** (3)

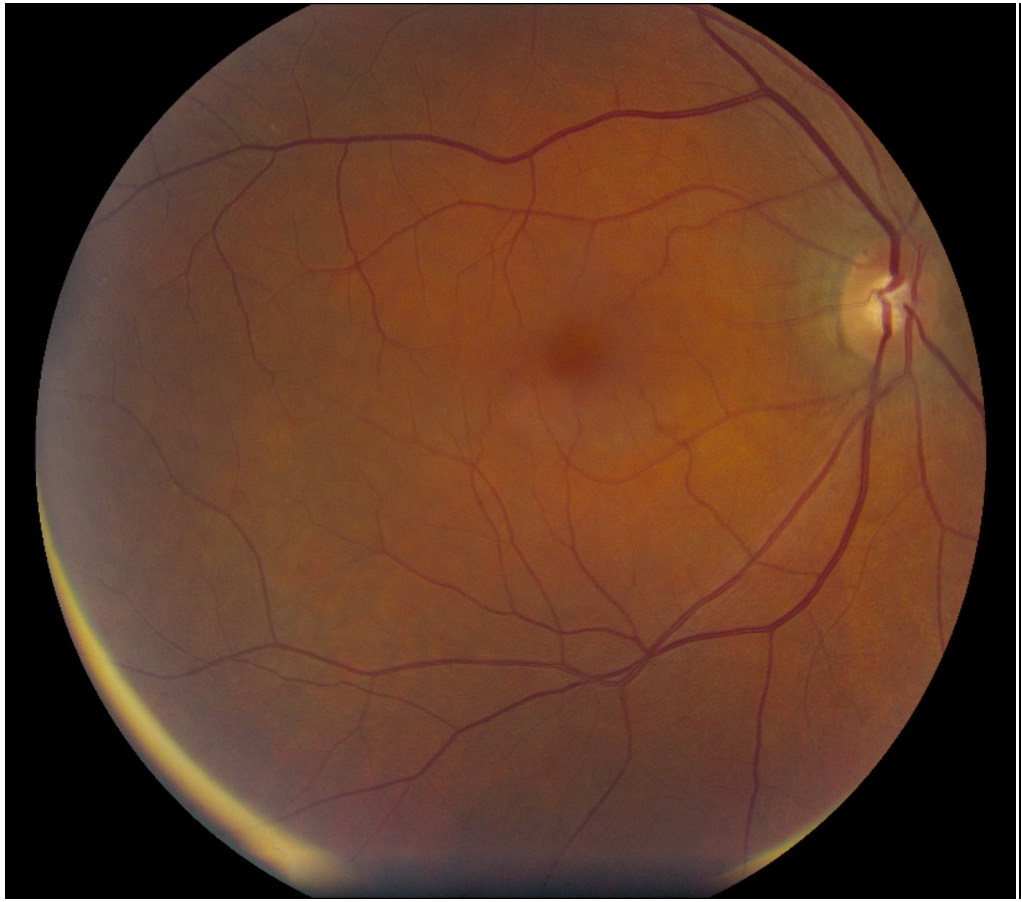

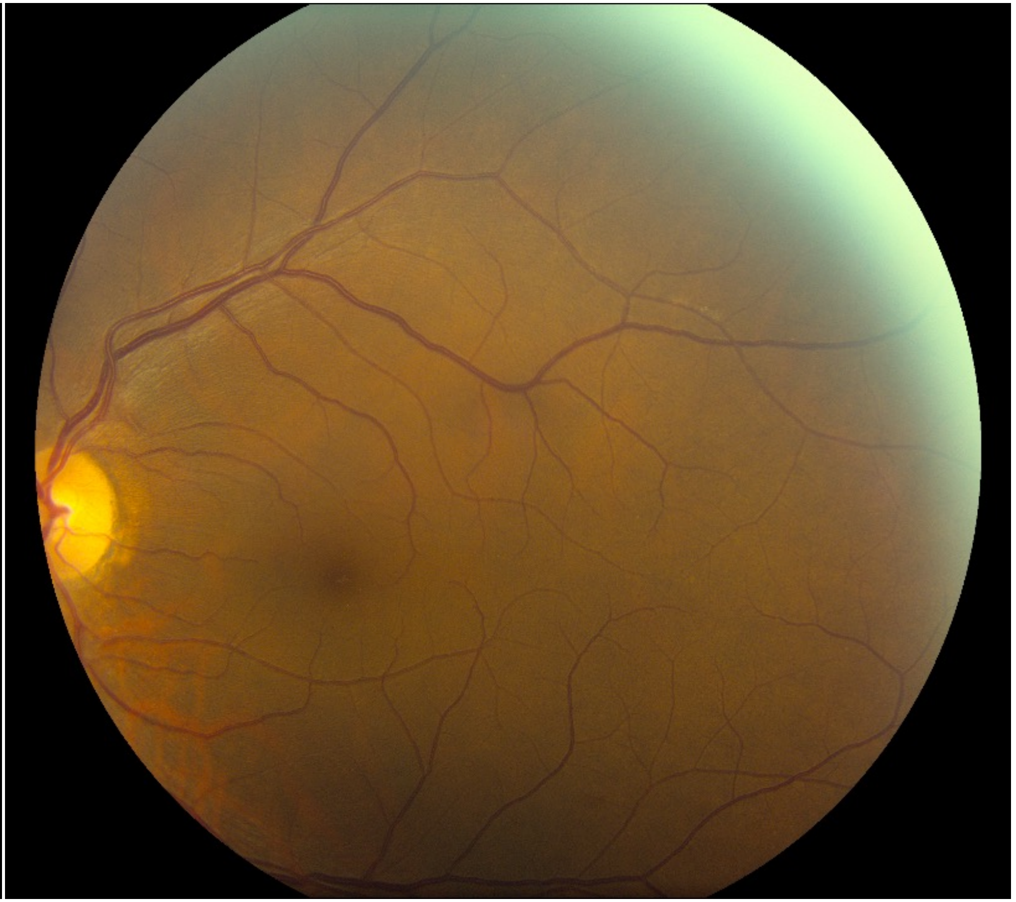

Insufficient for full interpretation – only parts of the retina recognizable

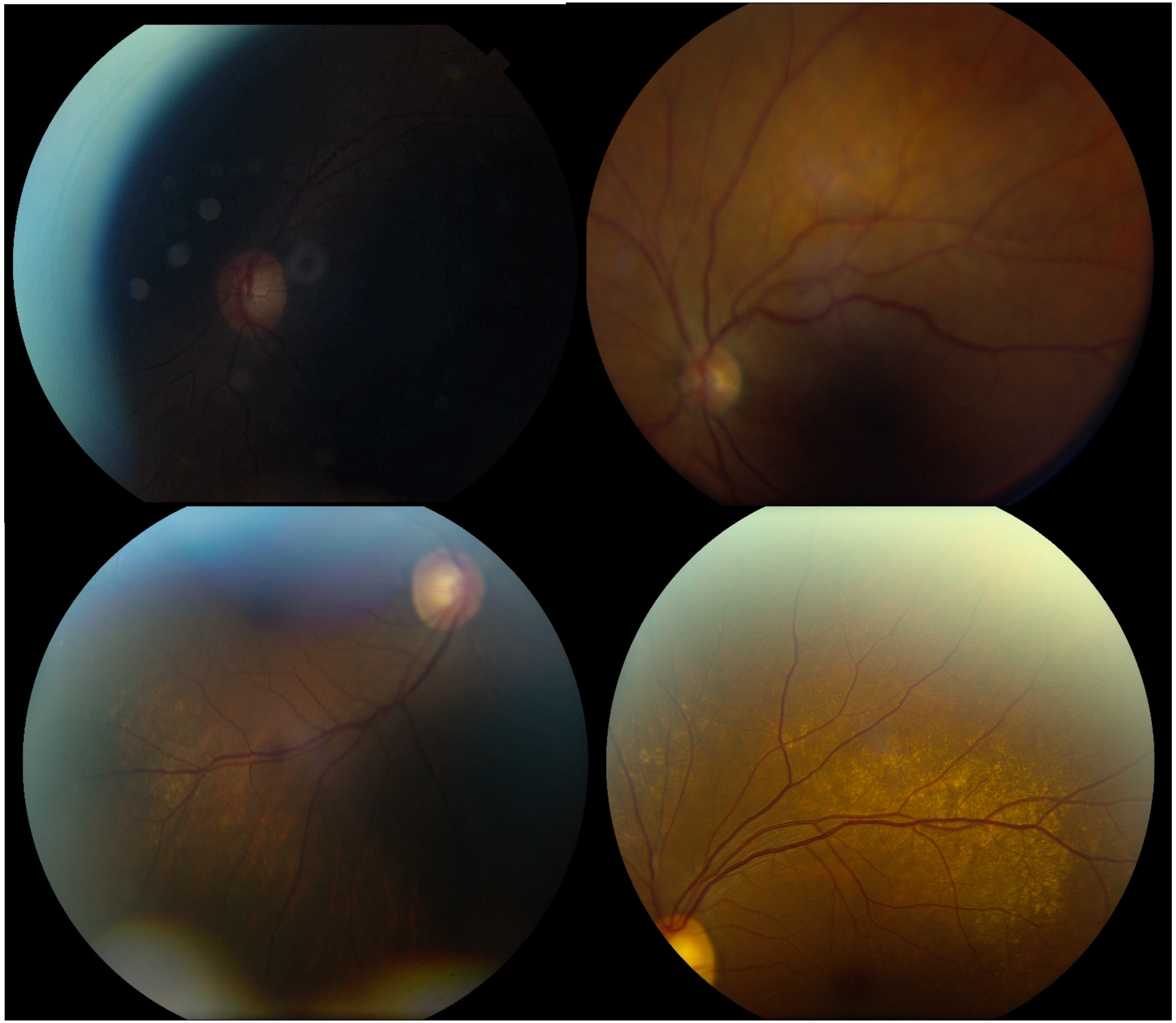

The grading tool

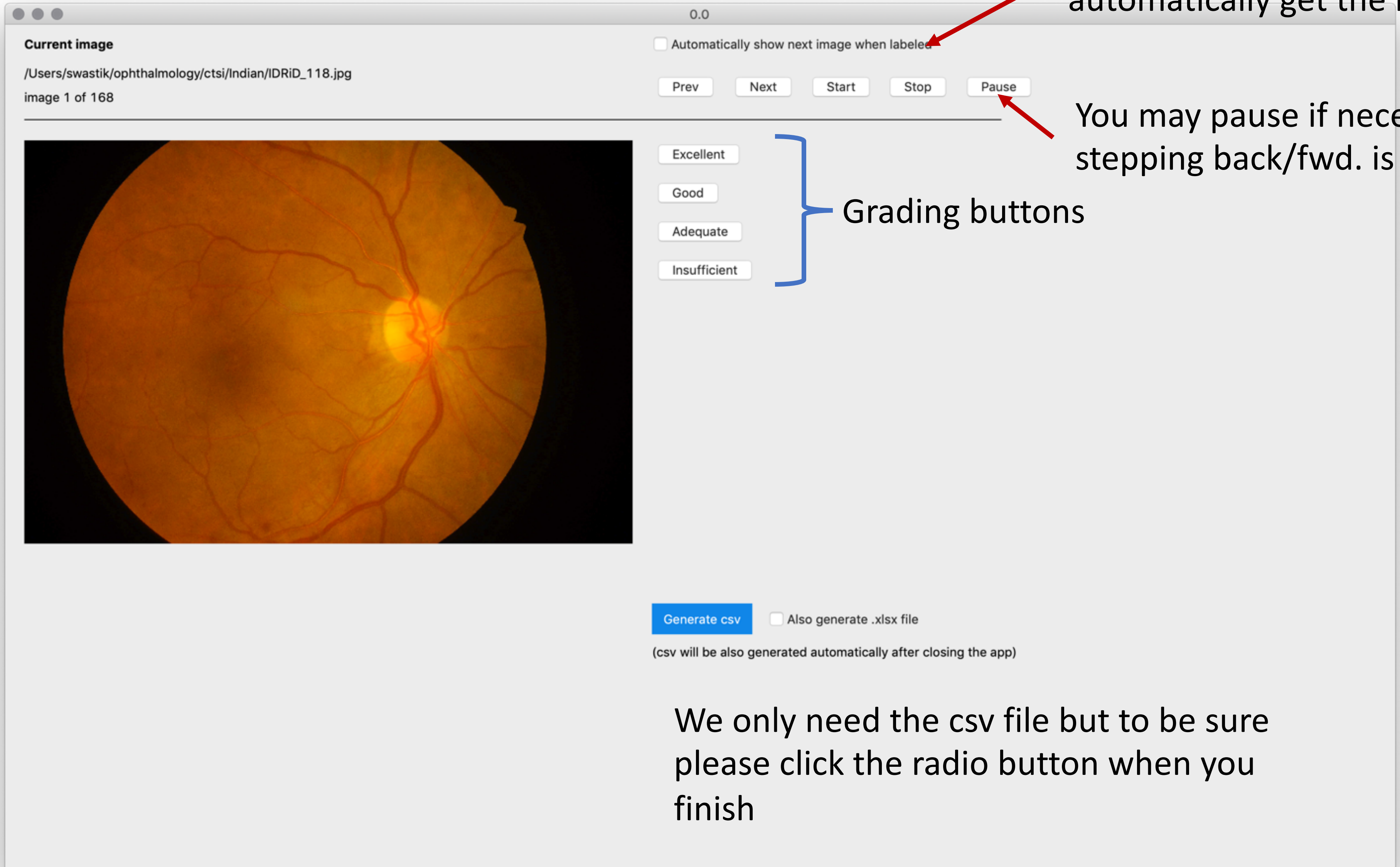

Check this box so that you automatically get the next image

You may pause if necessary, stepping back/fwd. is disabled

Grading buttons

We only need the csv file but to be sure please click the radio button when you finish

- During the grading feel free to leave the slidedeck with the grading description open so that you can check back in case you are insecure

Objective Image Grading, second round

Dear Participant,

We would like to cordially thank you for your time and effort to help us with this second round of grading using the instructions below:

The goal for this supplementary task is to try to increase the objectivity of the task.

You will be asked to grade 200 retinal images

The aim is to assess the quality of each image using single labels and group them accordingly

For grading you need to do the following

1. Similarly to the first round, assess each image for the 4 image quality factors:

    1. Focus
    2. Illumination
    3. Image field definition
    4. Artefacts

1.1. Examine the focus

Is the focus good enough for grading smaller retinal alterations (e.g. microaneurysms, venous beading or intraretinal microangiopathy)? Are small retinal vessels approximately one optic disk diameter around the fovea depicted sharply? If third generation branches within one optic disk diameter near the fovea can not be identified, the images should be considered inadequate.

2.1 Grade the image

Choose one of the buttons:

focus_optimal

focus_unsharp focus

1.2. Examine the illumination

Is the image too dark, or too light – overexposed? Are there dark areas or washed-out areas that interfere with detailed grading?

2.2. Grade the image

Choose one of the buttons:

illumination_optimal

illumination_too dark

illumination_too light

1.3. Examine the image field definition

Does the primary field include the entire optic nerve head and macula? Are the nasal and temporal fields adequately centered to include at least 80% of the non-overlapping portion of the field (i.e., the nasal portion of the nasal field, temporal portion of the temporal field)?

2.3. Grade the image

Choose a button:
Image field definition_optimal,
Image field definition_missing macula,
Image field definition_missing optic nerve head

1.4. Examine the artefacts

Is the image sufficiently free of artefacts (e.g. dust spots, arc defects, flashes, fingerprints or camera reflexes and eyelash images) to allow adequate grading

2.4. Grade the image

Choose one or moreof the buttons:

No artefacts

Artefacts_small pupil

Artefacts_dust spots

Artefacts_lashes

Artefacts_camera artefacts

Artefacts_arc defects

The grading tool

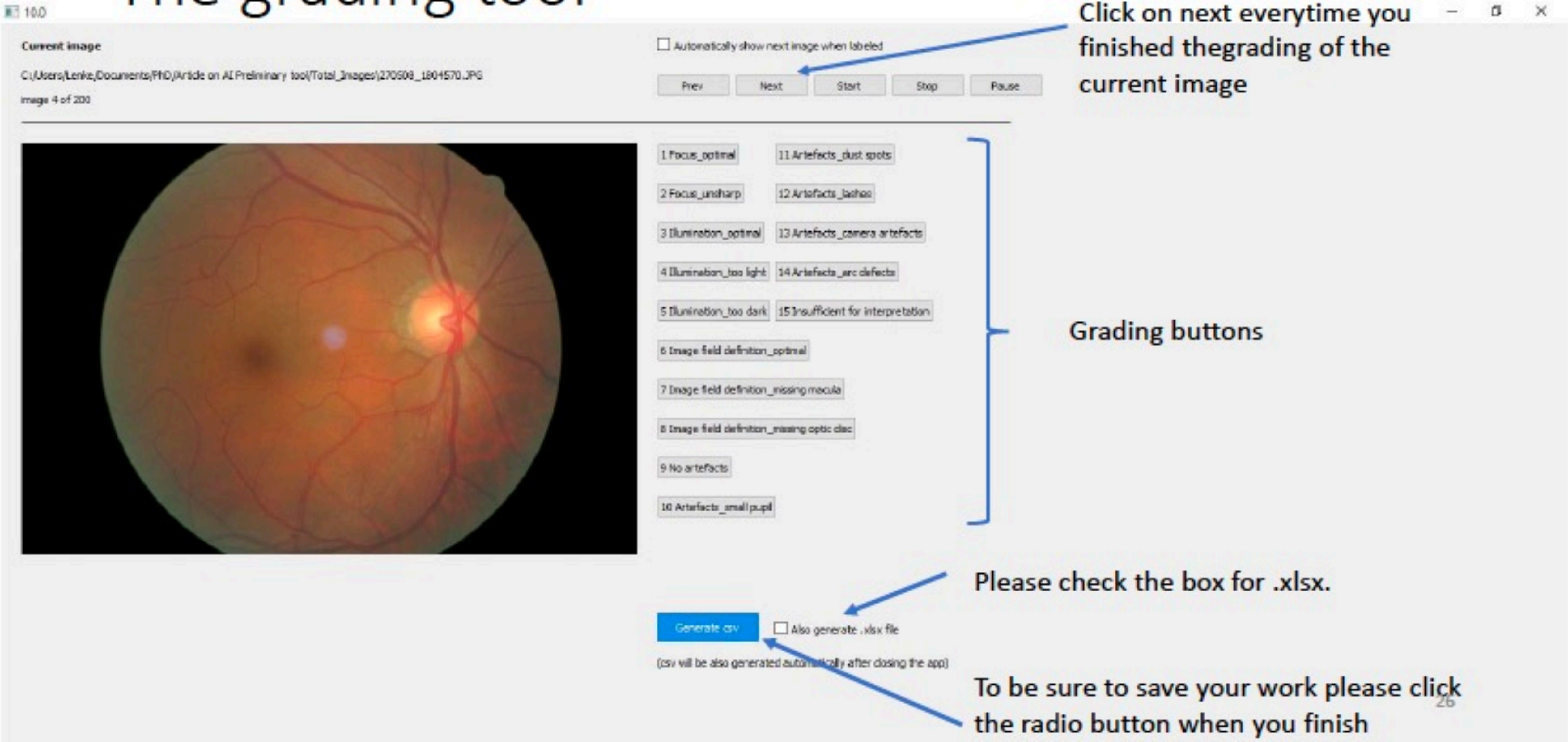

Click on next everytime you finished thegrading of the current image

Grading buttons

Please check the box for .xlsx.

To be sure to save your work please click the radio button when you finish

Once again we would like to thank you for your time and devotion

GOOD LUCK and ENJOY!

Lenke and Gábor
